# Effect of morpholine and charge distribution of cyanine dyes on cell internalization and cytotoxicity

**DOI:** 10.1038/s41598-022-07533-5

**Published:** 2022-03-09

**Authors:** Sirilak Wangngae, Kantapat Chansaenpak, Oratai Weeranantanapan, Pornthip Piyanuch, Thitima Sumphanapai, Montarop Yamabhai, Parinya Noisa, Rung-Yi Lai, Anyanee Kamkaew

**Affiliations:** 1 School of Chemistry, Institute of Science, Suranaree University of Technology, Nakhon Ratchasima, 30000 Thailand; 2 National Nanotechnology Center, National Science and Technology Development Agency, Thailand Science Park, Pathum Thani, 12120 Thailand; 3 School of Preclinical Sciences, Institute of Science, Suranaree University of Technology, Nakhon Ratchasima, 30000 Thailand; 4 School of Biotechnology, Institute of Agricultural Technology, Suranaree University of Technology, Nakhon Ratchasima, 30000 Thailand

**Keywords:** Chemistry, Chemical biology, Medicinal chemistry, Organic chemistry

## Abstract

To improve the potency of Heptamethine cyanines (Hcyanines) in cancer research, we designed and synthesized two novel Hcyanines based theranostic probes, **IR794-Morph** and **IR794-Morph-Mpip**, to enhance cancer cell internalization and targeting. In acidic conditions that resemble to tumour environment, both **IR794** derivatives exhibited broad NIR absorption band (704‒794 nm) and fluorescence emission (798‒828 nm) that is suitable for deep seated tumour imaging. Moreover, in vitro study revealed that **IR794-Morph-Mpip** exhibited better cancer targetability towards various cancer cell lines under physiological and slightly acidic conditions compared to normal cells. **IR794-Morph-Mpip** was fast internalized into the cancer cells within the first 5 min and mostly localized in lysosomes and mitochondria. In addition, the internalized signal was brighter when the cells were in the hypoxic environment. Furthermore, cellular uptake mechanism of both **IR794** dyes, investigated via flow cytometry, revealed that endocytosis through OATPs receptors and clathrin-mediated endocytosis were the main routes. Moreover, **IR794-Morph-Mpip**, displayed anti-cancer activity towards all tested cancer cell types with IC_50_ below 7 μM (at 6 h incubation), which is approximately three times lower than that of the normal cells. Therefore, increasing protonated cites in tumour environment of Hcyanines together with incorporating morpholine in the molecule can enhance structure-inherent targeting of these dyes.

## Introduction

Heptamethine cyanines (Hcyanines) are near-infrared (NIR) fluorescent dyes that can absorb and emit light in a range 700–1000 nm, which is suitable for tumour detection at millimetre depth due to less background fluorescence from endogenous molecules^1,2^. Hcyanines were used extensively as tools for cancer imaging because of their bright fluorescence in NIR region and good biocompatibility^3,4^. Moreover, some Hcyanines with a meso-chloride and a cyclohexenyl skeleton were found to accumulate and persist in solid tumors for several days^5–9^. Although there is no clear reason why they tend to accumulate in solid tumors, uptake into cancer cells via organic anion transporting polypeptides (OATPs)^10–12^ and roles of albumin in Hcyanines accumulation and persistence in solid tumor were discussed^6,13,14^. The uptake of Hcyanines in cancer cells was concerted actions exerted by hypoxia and activation of HIF1alpha/OATPs signalling leading to enhance dye uptake^9,15^. In general, cancer cells behave differently in comparison with normal cells^16,17^. For example, the lack of oxygen in tumour creates strong hypoxic condition leading to lactic acid build-up and lower extracellular pH level in tumour environment (pH 6.2–6.9)^18,19^. In addition, the lysosomal pH in cancer cells (pH_lys_ 3.8 – 4.7) expresses higher acidity than that in the normal cells (pH_lys_ 4.5–6.0)^20^.

Recently, an idea of developing structure–inherent targeting (SIT) NIR fluorescent dyes was proposed^5,21,22^. This offers a new opportunity to realize targeting delivery with no need of extra conjugations. In this work, we took an advantage of Hcyanines structures that can be recognized by OATPs on tumour cell surface. Additionally, we added more protonation sites into the structures to turn them to cationic probes when they reach tumour environment. Generally, particles carrying positive charges tend to have high cell-membrane binding affinity due to attractive electrostatic forces between cationic probe and anionic cellular membrane^23,24^. Therefore, we expected that our cationic dyes would internalize cancer cells faster than the neutral dyes.

Besides the charges, morpholine was also conjugated to the dyes aiming at lysosome targeting, which lysosomes emerged as an attractive target for cancer diagnosis and therapy^25,26^. Morpholine is a pharmacophore that involves in wide range of biological activities, including anti-cancer^27,28^. Altering pH in lysosomes might cause the organelle swelling and disruption, leading to cell death. Hence, if the particles could enter the cells via endocytosis, it has a high possibility to accumulate at lysosomes and trigger cellular cascades that can be harmful to the cells^29^.

Endocytosis is an energy-dependent process that cells transport substances from outside by engulfing them in a vesicle^30^. Large particles tend to be internalized by phagocytosis, whereas small molecules that suspended in extracellular fluid enter the cells via pinocytosis^31^. Pinocytosis including macropinocytosis, and clathrin- or caveolin-dependent endocytosis have been extensively studied^32,33^. Clathrin-mediated endocytosis plays a key role in cell signalling through the trafficking of membrane receptors^34^. After endocytosis, the uncoated vesicles will fuse with early endosomes and transport to Golgi for signal processing or direct to lysosomes for degradation while some of the receptors will recycle back to the membrane^35^. The clathrin-independent endocytosis, however, is much less understood, except caveolar endocytosis that is mediated by bulb-shaped plasma membrane named caveolae^33,36^.

Therefore, in this work, we developed a Hcyanine based theranostic probe (**IR794**), with NIR fluorescence and anti-cancer effect, that was intended to target tumour environment by selective internalization to cancer cells via OATPs. Our experimental data revealed that **IR794-Morph** and **IR794-Morph-Mpip** increased HepG2 cells uptake via clathrin-mediated endocytosis and offered high photocytotoxic efficacies. Moreover, **IR794-Morph-Mpip** (more positive charge) exhibited intense NIR absorbance in acidic pH and bright fluorescence in tumour cells especially under hypoxic environment. In addition, cytotoxicity profiles of these probes were also investigated in various cell lines.

## Results and discussion

### Synthetic procedures

**Figure 1:**
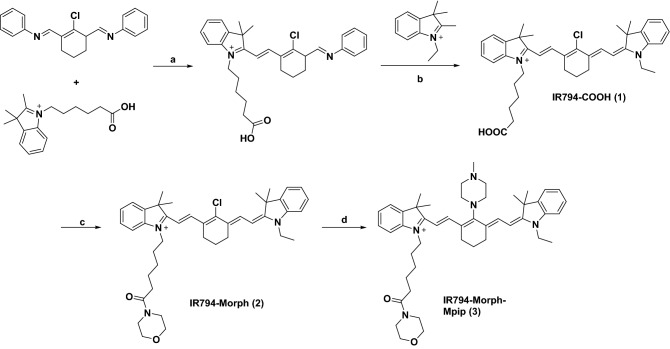
Syntheses of **IR794** derivatives. Reagents: (**a**, **b**) NaOAc, EtOH, 80 °C, 2 h^37^. (**c**) Morpholine, EDC.HCl, DMAP, CH_2_Cl_2_, 0 °C, under N_2_, 2 h^38^. (**d**) *N*-methylpiperazine, DMF, 25 °C, under N_2_, 3 h^39^.

Synthesis of asymmetrical Hcyanines **IR794-COOH (1)** involves the condensation of alkyl indoleninium salts in steps *a* and *b* (Fig. 1). **IR794-Morph (2)** was further synthesized via an amide coupling reaction between **IR794-COOH (1)** and morpholine using EDC.HCl as a coupling reagent and DMAP as a catalyst. **IR794-Morph-Mpip (3)** was synthesized by conjugation addition of Hcyanine **2** with *N*-methylpiperazine. These compounds were fully characterized by ^1^H and ^13^C NMR spectroscopy and high-resolution mass spectrometry (HRMS) (data are available in ESI).

### Photophysical properties

We expected that substitution of *N*-methylpiperazine group on the cyclohexenyl position of **2** yielded **3** with pH responsive ability. At neutral pH, the fluorescence of **3** is expected to be quenched by the effect from the nitrogen lone pair electrons of *N*-methylpiperazine moiety through a photoinduced electron transfer (PeT) process. While in acidic environments, protonation of the nitrogen atoms will block the PeT process causing the increased fluorescence signal. To explore this phenomenon, optical properties of **IR794** have been systematically investigated.

The absorbance and fluorescence spectra of **IR794** (**1–3**) were first measured in various organic solvents including, DMSO, MeCN MeOH, and EtOH. The photophysical properties listed in Table 1 showed that all three **IR794** compounds exhibited absorption maxima at the range from 704 to 794 nm while possessed emission maxima at the range from 798 to 828 nm. Interestingly, most of the stokes shifts of these compounds are in the scope from 28 to 65 nm, except that of **IR794-Morph-Mpip** in DMSO that displays a large stoke shift of 108 nm which could be the result from unique intermolecular forces between the methylpiperazine moiety and DMSO molecules. The fluorescent quantum yields (Φ_f_) of these compounds were obtained in the range between 0.1 and 0.2 which are typical among Hcyanines. However, it is worth to note that the probes in polar aprotic solvents (i.e. DMSO and MeCN) exhibited higher Φ_f_ than those in polar protic solvents (i.e. MeOH and EtOH).

**Table 1:** The photophysical properties of **IR794 (1–3)** in the different solvents.

Dye	Solvent	^a^λ_max_ (nm)	^b^λ_emiss_ (nm)	^c^∆ʋ(nm)	^d^*Φ*_*f*_	^e^*ε* (M^−1^ cm^−1^)
**IR794-COOH**	DMSO	794	827	33	0.192	5.1 × 10^4^
	MeCN	779	808	29	0.202	3.4 × 10^4^
	EtOH	784	812	28	0.164	5.0 × 10^4^
	MeOH	779	809	30	0.119	6.7 × 10^4^
**IR794-Morph**	DMSO	794	828	34	0.176	7.8 × 10^4^
	MeCN	779	810	31	0.149	7.5 × 10^4^
	EtOH	783	812	29	0.166	7.9 × 10^4^
	MeOH	779	808	29	0.120	8.6 × 10^4^
**IR794-Morph-Mpip**	DMSO	704	812	108	0.206	2.2 × 10^4^
	MeCN	745	801	56	0.167	3.3 × 10^4^
	EtOH	743	800	57	0.131	3.5 × 10^4^
	MeOH	733	798	65	0.101	3.7 × 10^4^

^a^λ_abs_ = absorption maximum wavelength, ^b^λ_em_ = emission maximum wavelength (Excitation wavelength = 750 nm), ^c^∆λ = stokes shifts (λ_em_ − λ_abs_), ^d^*Φ*_f_ = fluorescence quantum yields calculated by using Indocyanine green (ICG) was used as a standard (*Φ* = 0.13 in DMSO), ^e^*ε* = molar absorptivity.

**Figure 2:**
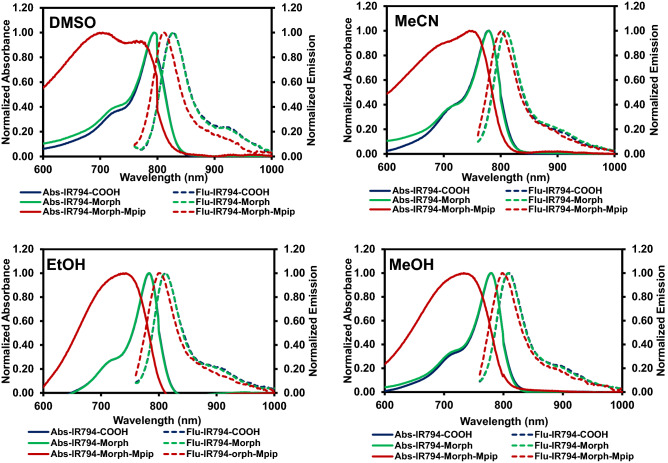
Vis–NIR absorption and fluorescent spectra of **IR794** derivatives (1 µM) excited at 750 nm in DMSO, MeCN, EtOH and MeOH.

Based on the absorption and emission spectra demonstrated in Fig. 2, **IR794-COOH** and **IR794-Morph** displayed inherent spectral characteristics of the typical Hcyanines in all solvents. Notably, the absorption spectra of **IR794-Morph-Mpip** showed a broad appearance which could be the effect from *N*-methylpiperazine moiety as seen in our previous report^39^. The excitation spectra of **IR794-Morph-Mpip** in organic solvents also demonstrated similar appearance with the absorption spectra as presented in Fig. S1.

### pH effects of IR794 by vis–NIR and fluorescence spectroscopic analysis

**Figure 3:**
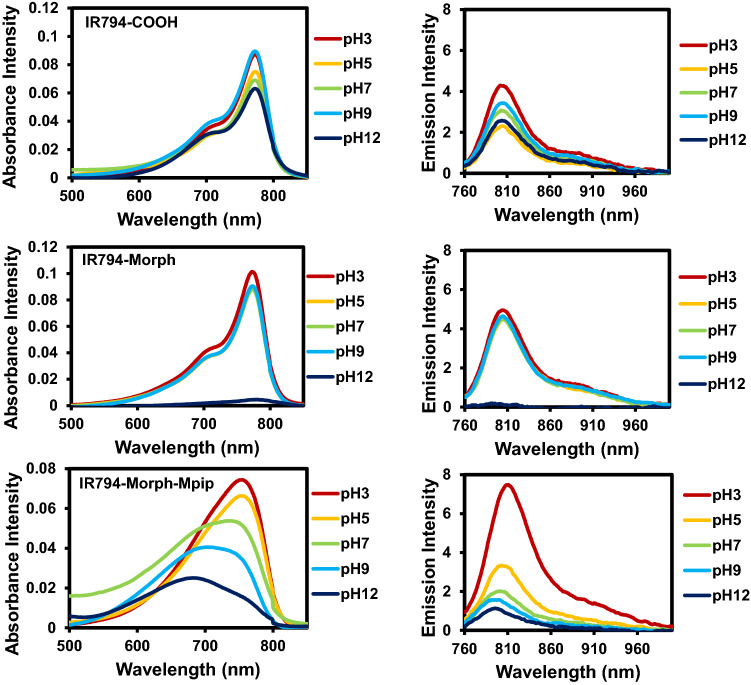
Vis–NIR absorption and fluorescent spectra of **IR794** in different pH 3.0–12.0.

Since the **IR794** derivatives contain different functional groups in the structures (‒COOH, ‒Morph and –Morph-Mpip), it is possible that they could form different charge states at different pH (3–12), leading to the alteration of photophysical properties of our **IR794**. As shown in Fig. 3, there is no significant changes in **IR794-COOH** absorption and emission spectra under either acidic or basic conditions. In contrast, **IR794-Morph**, shows hypochromic effect of the spectra where the absorption and emission decreasing the intensity in highly basic condition (pH 12).

**IR794-Morph-Mpip**, on the other hand, displays bathochromic shifts of absorbance and fluorescence spectra in acidic conditions. In addition, the emission signals are enhanced as the acidity of the media increases. These phenomena might cause by the effect of proton exchange of *N*-methylpiperazine moiety (pK_a_ = 3.81 and 8.38)^40^ that alter optical properties of the dye subjectable to the pH of the solutions. **IR794-Morph-Mpip** absorbs light from visible to NIR region (500–920 nm), peaking at 750 nm under neutral pH. When two nitrogen atoms of *N*-methylpiperazine on **IR794-Morph-Mpip** are fully protonated under acidic environments (pH 3, 5), absorption spectra are red shifted. Whereas, once **IR794-Morph-Mpip** exposes to basic environments (pH 9, 12), absorption spectra are blue shifted. These could be explained by an intramolecular charge transfer (ICT) within the molecule. Since the **IR794-Morph-Mpip** contains both electron donor (amine) and acceptor (Hcyanine), a charge separation is obtained within the fluorophore. The electron-donating ability of the donor at the meso-position in a cyanine scaffold would cause absorption and/or fluorescence spectra shift as seen in the previous study^39^.

**Figure 4:**
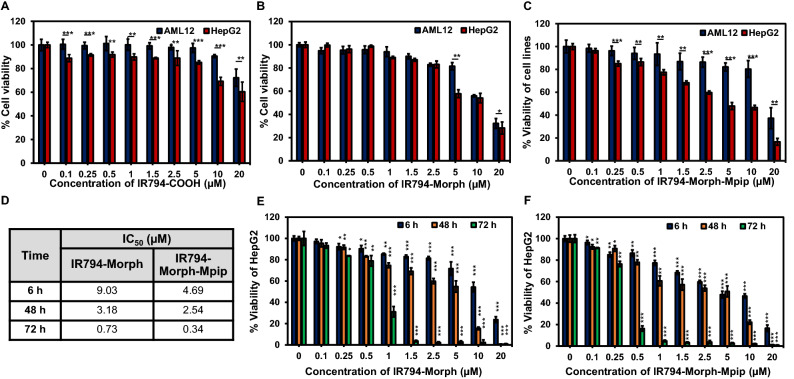
Relative viabilities of AML12 cells (blue bars) and HepG2 cells (red bars) after exposure to **IR794-COOH** (**A**), **IR794-Morph** (**B**) and **IR794-Morph-Mpip** (**C**) at various concentrations (0‒20 μM) for 6 h. (**D**) IC_50_ values of **IR794-Morph** and **IR794-Morph-Mpip** treated with HepG2 for 6, 48 and 72 h. Relative viabilities of HepG2 cells treated with **IR794-Morph** (**E**) and **IR794-Morph-Mpip** (**F**) for 6 h (blue bars), 48 h (orange bars) and 72 h (green bars). Statistical analysis is based on Paired Student’s T-test analysis (*P < 0.05, **P < 0.01, ***P < 0.001) where (**A**–**C**) is the comparison between AML-2 and HepG2 at the same concentration; (**E**,**F**) is the comparison between the treatment and the control.

To discover applications of **IR794** derivatives in living cells, series of in vitro experiments were performed. First, cytotoxicity profile of **IR794** on liver cells including normal, alpha mouse liver 12 (AML12), and cancer, human hepatoma (HepG2), cells were evaluated at various concentrations of **IR794** (0‒5.0 μM) for 6 h. The comparative cell viability was determined by standard MTT assays^39,41^. As shown in Fig. 4A–C, at concentrations up to 5 μM, the normal cells maintained more than 80% viability when they exposed to **IR794** derivatives. However, the compounds started to cause toxicity to the normal cells when the dose were higher than 5 μM. Remarkably, among the series, **IR794-Morph-Mpip** exhibited the best cancer selectivity with significant anti-cancer effect towards HepG2 with IC_50_ 4.69 μM (IC_50_ = 8.88 μM for **IR794-Morph** and IC_50_ > 20 μM for **IR794-COOH**, Table 2 and Fig. S2).

**Table 2:** IC_50_ of **IR794** treated with AML12 and HepG2 cells for 6 h.

Cells	IC_50_ (µM)		
	**IR794-COOH**	**IR794-Morph**	**IR794-Morph-Mpip**
AML12	> 20	12.01	19.68
HepG2	> 20	8.88*	4.69*

Moreover, to better understand the effect of the compounds on cell viability, HepG2 cells were incubated with **IR794-Morph** and **IR794-Morph-Mpip** for extended period (48 and 72 h). As expected, a prolonged incubation resulted in reducing cell viability (Fig. 4D–F). After 72 h treatment, HepG2 cell viability reduced dramatically when exposed to **IR794-Morph** and **IR794-Morph-Mpip** with IC_50_ = 0.73 and 0.34 μM, respectively (Figs. 4D and S2). However, the cytotoxicity of these two probes is still not as good as that of an anti-cancer agent, *i.e.* doxorubicin, which has IC_50_ = 2.16 and 0.02 μM when the cells were treated for 6 and 24 h, respectively (Fig. S3). Nevertheless, **IR794-Morph-Mpip** displayed superior anti-cancer effect compared to **IR794** analogues.

**Figure 5:**
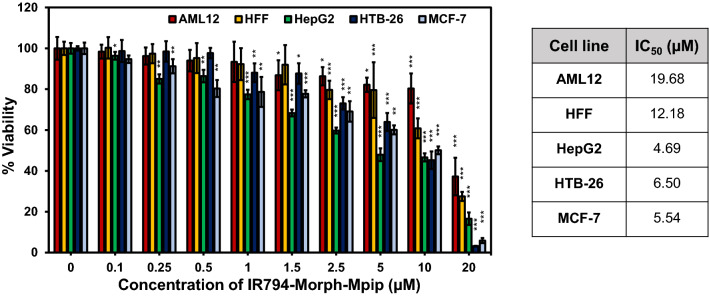
Relative viabilities of various cell lines after exposure to **IR794-Morph-Mpip** at various concentrations (0‒20 μM) for 6 h. Statistical analysis is based on Pair Student’s T-test analysis (*P < 0.05, **P < 0.01, ***P < 0.001) which compared between the treatment and the control of each cell line. Table shows IC_50_ of **IR794-Morph-Mpip** treated on different cell lines.

As **IR794-Morph-Mpip** showed some cancer selectivity, its activity towards other cell lines including human foreskin fibroblasts (HFF), and human breast cancer cells (HTB-26 or MDA-MB-231 and MCF-7) was also investigated. After 6 h incubation, **IR794-Morph-Mpip** exhibited selective cancer eradicating with IC_50_ values below 7 μM for all cancer lines, whereas IC_50_ values are higher than 12 μM for the normal lines (Fig. 5). However, after extended incubation time, no significant cancer cell selectivity was observed.

### Cell internalization

**Figure 6:**
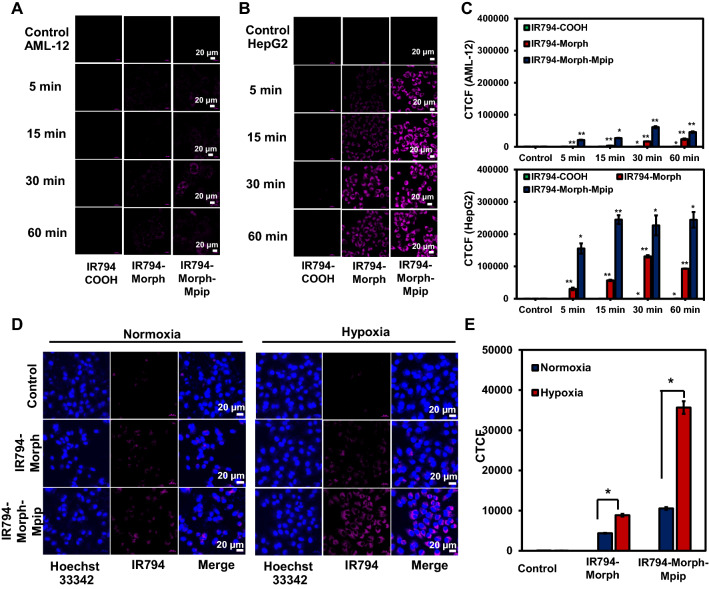
Confocal images of (**A**) AML12 and (**B**) HepG2 cells obtained using a laser scanning confocal microscope (Nikon A1Rsi, 63 × oil immersed optics) incubated with 1 μM of **IR794** for 5–60 min. (**C**) Quantitative fluorescent intensity represented as corrected total cell fluorescence (CTCF), which were quantified using ImageJ and represent the mean ± SD (from three independent experiments, 30 cells/set). (**D**) Confocal images of HepG2 cells incubated with 0.5 μM of **IR794-Morph** and **IR794-Morph-Mpip** for 5 min under normoxia and hypoxia conditions. (**E**) Quantitative CTCF quantified using ImageJ and represent the mean ± SD (n = 3, 30 cells/set). Statistical analysis is based on T-test (*P < 0.05, **P < 0.01, ***P < 0.001) where the comparison between the control and different incubation duration (for each compound) is marked in C and the comparison between normoxia and hypoxia is marked in (**D**). Scale bar = 20 μm.

Subsequent, time dependent internalization of **IR794** in normal cells (AML12) and cancer cells (HepG2) was monitored to investigate how fast the probes can enter cells. At the first hour, there was little to no internalized signals of all three probes from AML12 cells (Figs. 6A–C and S4). In contrast, the fast uptake of **IR794-Morph-Mpip** in HepG2 cells was obviously detected within the first 5 min of incubation (Figs. 6B,C and S5). In case of **IR794-Morph,** the signal was clearly observed from HepG2 cells after 15 min incubation, but the intensity was not as bright as the fluorescence from **IR794-Morph-Mpip**. Interestingly, there is no signal of **IR794-COOH** from both normal and cancer cells, implying no uptake of this dye at the first hour of incubation. In addition, **IR794-Morph-Mpip** was found to be fast internalized in all tested cancer cell lines (MCF-7, HTB26 and HepG2), Fig. S6.

To further verify that **IR794-Morph-Mpip** is a pH-sensitive theranostic reagent and the increasing of protonation sites could improve the targeting of cyanine dyes to tumour cells, cellular uptake under hypoxia condition was investigated. As depict in Fig. 6D and E, compared to the normoxic condition, the fluorescent signals significantly enhance when **IR794-Morph-Mpip** treated with hypoxic cells only for 5 min at low dose (0.5 μM). Nevertheless, **IR794-Morph** did not show the same effect under hypoxia. Tumour hypoxia is a common feature in solid tumour that arises when oxygen supply is deficient, resulting in an acidic environment inside tumour micro-environment^19^. Therefore, when **IR794-Morph-Mpip** reaches hypoxic environment, it is highly protonated leading to fluorescence enhancement.

**Figure 7:**
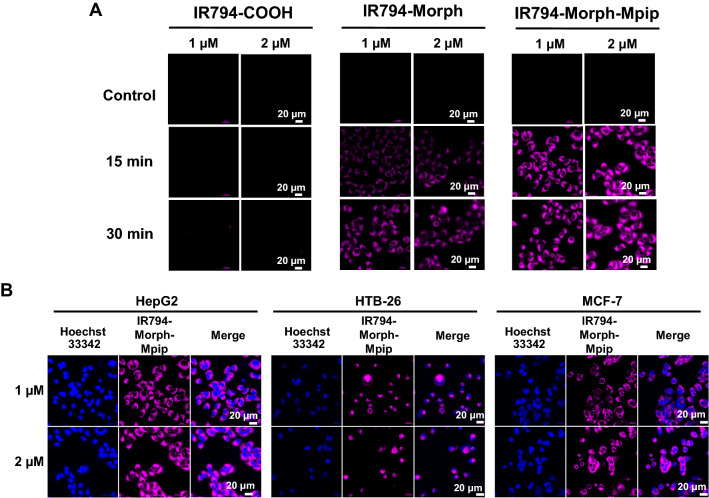
(**A**) Confocal images of dose-dependent effect of **IR794** (1 and 2 µM) in HepG2 cells, which were incubated for 15 and 30 min. (**B**) Confocal images of cancer cells (HepG2, HTB-26 and MCF-7) after treated with **IR794-Morph-Mpip** (1 and 2 µM) for 15 min.

Furthermore, in HepG2 cells, the fluorescence signal was found to be in a dose-dependent manner (Fig. 7A). When concentrations of **IR794-Morph-Mpip** and **IR794-Morph** increased from 1 to 2 µM, the HepG2 cells uptake was also improved. After incubation time was increased to 30 min, the enhancement of the signal was also observed. The difference is more noticeable in the case of **IR794-Morph** (15 min *vs* 30 min), while signal from **IR794-Morph-Mpip** seems to be saturated since 15 min incubation. Moreover, the signal from **IR794-Morph-Mpip** in other cancer cells (MCF-7 and HTB-26) was slightly higher when the dose increased (Fig. 7B); quantitative fluorescent signals are shown in Fig. S7. From this result, 1 µM of **IR794-Morph-Mpip** and **IR794-Morph** was enough for cell internalization and could be used as an indicator to distinguish cancer from normal cells.

### Intracellular localization study

**Figure 8:**
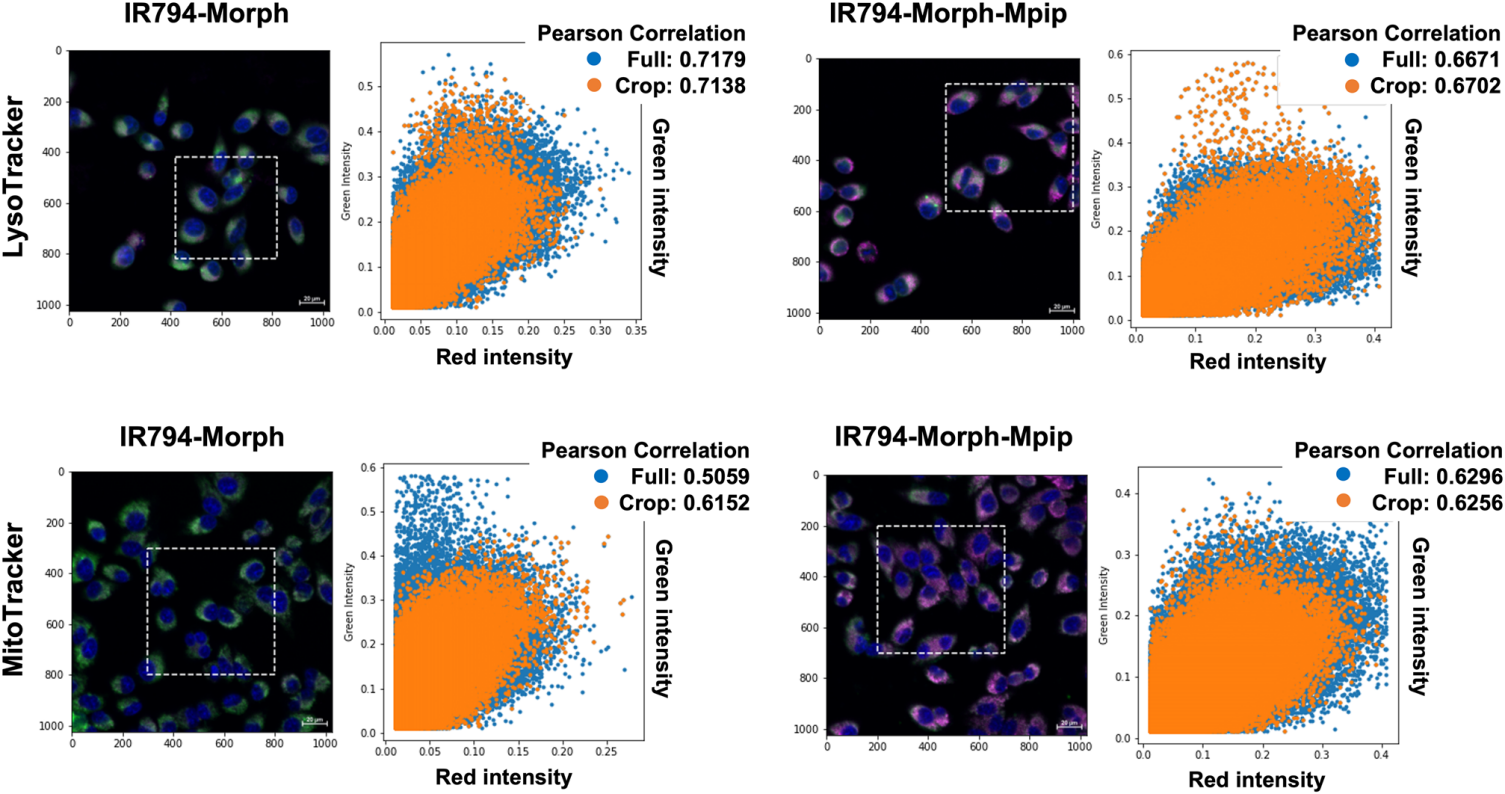
Confocal images of HepG2 cells incubated with 1 µM of **IR794-Morph** and **IR794-Morph-Mpip** for 15 min and colocalization of the probes with LysoTracker green (Pearson’s R value = 0.72 for **IR794-Morph** and 0.67 for **IR794-Morph-Mpip**) and MitoTracker (Pearson’s R value = 0.51 for **IR794-Morph** and 0.63 for **IR794-Morph-Mpip**). Pearson’s correlation coefficient between the two intensities was performed in python, using a built-in function from scipy statistical package.

As **IR794-Morph** and **IR794-Morph-Mpip** were fast uptake in cancer cells, intracellular localization of both probes was studied. Both dyes contain morpholine moiety in the structure, which is known to direct a molecule to lysosomes^42–44^. Moreover, some reported Hcyanines with net positive charge on the structures tend to localize in mitochondria^45,46^. Therefore, Hoechst 33342 (for nuclei), LysoTracker (for lysosomes) and MitoTracker (for mitochondria) were used as markers to pinpoint the intracellular localization of **IR794-Morph** and **IR794-Morph-Mpip**. The confocal images in Fig. 8 reveals magenta fluorescence of **IR794-Morph** and **IR794-Morph-Mpip** overlaps with LysoTracker (Pearson’s R value = 0.72 for **IR794-Morph** and 0.67 for **IR794-Morph-Mpip**) and MitoTracker (Pearson’s R value = 0.51 for **IR794-Morph** and 0.63 for **IR794-Morph-Mpip**). From these results, **IR794-Morph** prefers to localize in lysosome rather than mitochondria. Whereas **IR794-Morph-Mpip** localizes in both organelles with a similar amount. This could be because extra positive charge on the *N*-methylpiperazine makes the molecule favours import across the inner mitochondrial membrane.

### Cellular trafficking study

Furthermore, we would like to understand the uptake pathways of both dyes, **IR794-Morph** and **IR794-Morph-Mpip**. We hypothesized that the extra positive charges of **IR794-Morph-Mpip** might alter the uptake mechanism of the dye.

**Figure 9:**
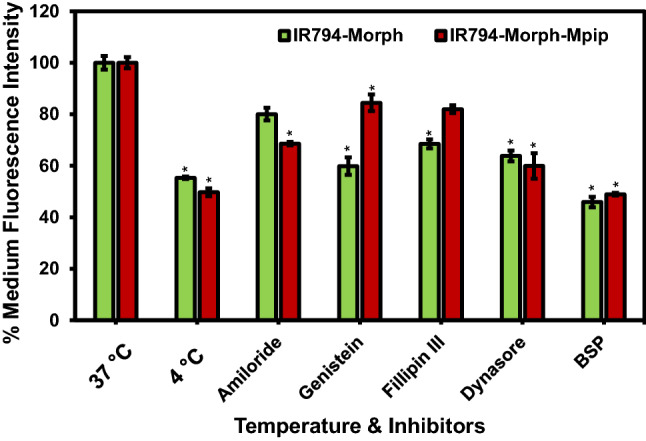
Flow cytometry of HepG2 cells incubated with 1 μM of **IR794** for 15 min in culture media. Cellular trafficking pathway of **IR794-Morph** and **IR794-Morph-Mpip** characterised by various inhibitors and at 4 °C condition. The endocytosis inhibitors used include amiloride (micropinocytosis), genistein (caveolin-mediated), Fillipin III, dynasore (clathrin-mediated) and BSP. Data reports as % Medium fluorescence intensity and represents the mean ± SD (n = 3, independent experiment). Statistical analysis is based on Pair Student’s T-test analysis (*P < 0.05, **P < 0.01, ***P < 0.001) where the comparison between the uptake at 37 °C and the uptake in the presence of different inhibitors is marked.

In general, cellular uptake of macromolecules occurs via ATP-dependent endocytosis and this process can be attenuated at low temperature. Therefore, the effect of temperature on cellular uptake was studied by incubating **IR794-Morph** and **IR794-Morph-Mpip** with HepG2 cells at 4 °C for 30 min and the cell uptake was quantified using flow cytometry. At 4 °C, percent medium fluorescence intensity (% MFI) of both compounds is about 50% (Fig. 9) compared to the uptake at 37 °C. This indicates that the endocytosis of both dyes is energy dependent.

To further explore cellular trafficking, a panel of endocytosis inhibitors were selected to inhibit specific endocytic pathways^47^. These include Filipin III and genistein, which inhibit caveolae-mediated endocytosis, amiloride which is against micropinocytosis, and dynasore which blocks clathrin-mediated endocytosis. Quantitative fluorescence from flow cytometry displayed that genistein, Filipin III, and dynasore had significant effect on the internalization of **IR794-Morph** by reducing the cell uptake to lower than 70%. However, amiloride and dynasore showed significant inhibition of **IR794-Morph-Mpip** uptake (< 70%). These findings suggested that both dyes follow endocytosis through clathrin-mediated pathway. In addition, caveolae-mediated endocytosis is a predominant pathway for **IR794-Morph** uptake whereas micropinocytosis is another main route for **IR794-Morph-Mpip** uptake.

**Figure 10:**
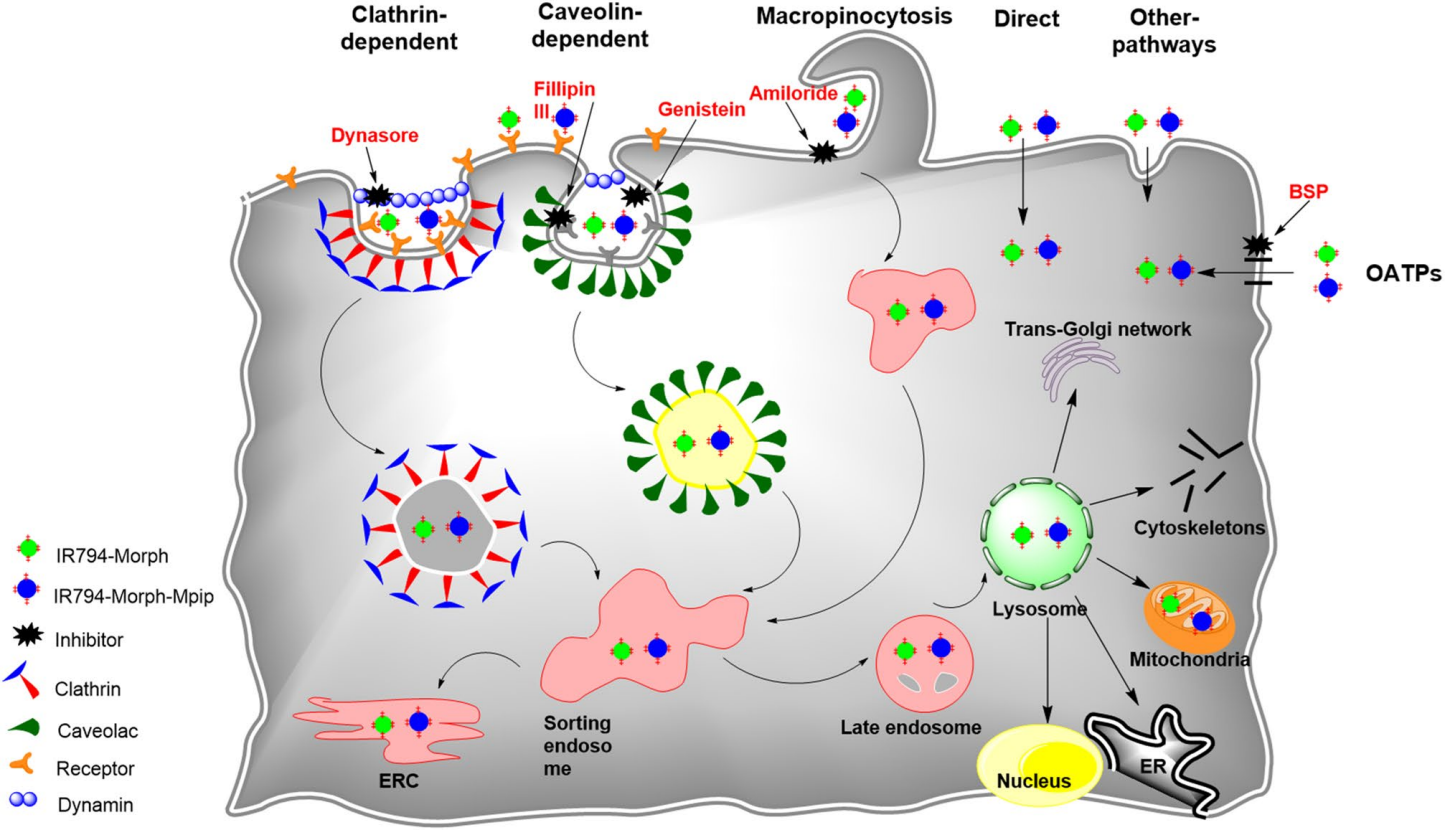
Schematic representation of the possible internalization pathways of **IR794-Morph** and **IR794-Morph-Mpip**.

It was reported that some Hcyanine dyes containing cyclohexyl ring in the conjugation system localize in solid tumor^48^ but not in normal tissue^12,45,49–51^ which the organic anion transporter proteins (OATPs) was suggested to be the main uptake channel. OATPs are the cell surface receptors that overexpressed on solid tumors but not on the normal tissue^52^. Therefore, in this study, we treated a nonspecific OATPs inhibitor, bromosulfophthalein (BSP), with HepG2 cells prior to add **IR794-Morph** and **IR794-Morph-Mpip** to determine if the dyes uptake and accumulation were dependent upon OATPs. From Fig. 8, after blocking OATPs, the uptake of both dyes was significantly reduced, confirming the OATP-dependent trafficking. Schematic representation of possible internalization pathways of our probes and inhibitors is displayed in Fig. 10.

## Conclusions

In summary, **IR794-Morph** and **IR794-Morph-Mpip** were successfully developed as pH-sensitive theranostic agents for fluorescent imaging and anti-cancer. **IR794-Morph-Mpip** absorbs slightly red-shifted in NIR region under acidic conditions and emit strong fluorescence at low pH, which is suitable to visualize cells in tumour environment, especially in hypoxia. Moreover, **IR794-Morph** and **IR794-Morph-Mpip** selectively internalized cancer cells and the fast uptake was observed in dose- and time-dependence manners. Cytotoxicity profiles confirmed selectivity of both probes toward cancer cells where the derivative containing morpholine, **IR794-Morph-Mpip**, offered superior anti-cancer activity with IC_50_ below 7 μM for all tested cancer cells. Intracellular trafficking of both probes was proved to be endocytosis via transporters (OATPs) and clathrin-dependent pathway. Therefore, adding morpholine moiety and increasing protonation sites of cyanine dyes enhance cancer cells selectivity and cytotoxicity.

## Methods

### General details for vis–NIR and fluorescence measurements

Stock solutions (1000 µM) of **IR794** probes were prepared in DMSO then diluted to 1 µM in a 3 mL quartz cuvette in various solvents (DMSO, MeCN, EtOH, MeOH and buffers pH 3–12). Vis–NIR absorption spectra were recorded on a UV–vis Spectrophotometer (Agilent Technologies Cary 300). The fluorescence spectra were recorded by PTI QuantaMaster 500—Near Infra-Red Photoluminescence System (HORIBA Scientific), using the following parameters: excitation wavelengths = 750 nm, excitation slit widths = 10 nm, and emission slit widths = 10 nm.

### Cell culture

Human liver hepatocellular carcinoma (HepG2), human breast cancer (MCF-7), human breast adenocarcinoma (HTB-26 or MDA-MB-231), human foreskin fibroblasts (HFF) and alpha mouse liver 12 (AML12) cell lines were purchased from the American Type Culture Collection (ATCC) and cultured according to the company procedure. All the cells were incubated at 37 °C in a humidified 95% air, 5% CO_2_ atmosphere.

### Cell viability assay

The cells were seeded roughly 7 × 10^4^ cells per well on 96-well plate and incubated in completed media for 24 h. Thereafter, the cells were treated with 0, 0.1, 0.25, 0.50, 1.0, 1.5, 2.5, 5.0, 10 and 20 µM of **IR794** series and continued culturing for 6, 48 and 72 h. After washing with PBS, the cell viability was determined using the standard MTT protocol^39,41^.

Relative viability of cell is calculated by: % Viability = (*A*_treatment_ − *A*_blank_)/(*A*_control_ − *A*_blank_) × 100% (where, *A* = absorbance at λ = 560 nm).

### Time- and dose-dependent internalization

Cells, approximately 1 × 10^4^ cells, were seeded on an 8-well chambered coverglass (LabTek, Nunc) and incubated in completed media for 24 h. For time dependent experiment, the cells were treated with 1 µM of **IR794** for 0, 5, 15, 30 and 60 min. For dose dependent experiment, the cells were treated with 1.0 and 2.0 µM of **IR794** probes for 15 and 30. Before imaging with Laser Scanning Confocal Microscope (Nikon A1Rsi), all the cells were washed with PBS and stained with Hoechst 33342. Excitation lasers: 641 nm (**IR794** probes); 405 nm (Hoechst 33342). A 60 × oil immersion objective lens was used. All data were analyzed by ImageJ and presented quantitative fluorescent intensity as corrected total cell fluorescence (CTCF), mean ± SD (n = 30).

For co-localization study, after cells were incubated with **IR794** probes, they were stained with Lysotracker Green DND 26 or Mitotracker Green FM (Thermo Fisher Scientific) for 20 min. Before visualization under LSCM, the cells were washed and stained with Hoechst 33342. To quantify the colocalization intensities of the red and green channels, each pixel of the confocal image, corresponding to the excitations of the probes and organelles trackers, was used in the calculation of Pearson’s correlation coefficient between the two intensities using python with a built-in function from scipy statistical package.

### Flow cytometry

Experiment was performed according to the previous protocol^39^. Briefly, the cells were seeded into 6-well cell culture plates density of 3 × 10^5^ cells/ well and incubated for 24 h. After media were removed, solutions of **IR794** in DMEM with final concentrations of 1 µM were added. After 1 h incubation, the cells were thoroughly washed with PBS and harvested by trypsinization then transferred into Eppendorf tubes (1.5 mL). Non-uptake dye was removed by washing with ice cold PBS and centrifugation at 800×*g*, 4 °C for 5 min, then resuspended and repeat washing for 3 times. Trypan blue (0.2%, 1 mL) in PBS was added to the cells to quench non-internalized signal before analysed by flow cytometry (Attune NxT Flow Cytometer, Life Technologies) using red excitation laser 637 nm and emission filter 780/60 nm.

### Cellular trafficking study

Temperature’s effect on cellular uptake was examined by incubating HepG2 cells treated with **IR794-Morph** and **IR794-Morph-Mpip** (1 µM) at 4 °C for 30 min. The endocytosis pathway was further investigated using different endocytosis inhibitors, including amiloride hydrochloride (final concentration 10 µM), genistein (100 µM), filipin III (2.5 µM) and dynasore (80 µM)^47^. HepG2 cells (3 × 10^5^ cells per well) in 6-well plate were pre-incubated with inhibitors individually for 30 min at 37 °C. Afterwards, treated cells were incubated with 1 µL of **IR794-Morph** and **IR794-Morph-Mpip** for 15 min. **IR794-Morph** and **IR794-Morph-Mpip** without inhibitors was used as controls. Data were analysed as mentioned above in “Flow cytometry” method.

### Statistical analysis

Data are expressed of three independent experiments (n = 3) and presented as the mean of at least four individual observations with the standard deviation (mean ± SD). The statistical analysis was performed using Paired Student’s T-test analysis. P-values < 0.05 were considered to indicate significance (*P < 0.05, **P < 0.01, ***P < 0.001).

## Supplementary Information

Supplementary Information.

## Data Availability

All data generated or analysed during this study are included in this published article and Supplementary Information file.
