# Supplementary material for: Effect of morpholine and charge distribution of cyanine dyes on cell internalization and cytotoxicity
Source: Sci Rep. 2022 Mar 9;12:4173. doi: 10.1038/s41598-022-07533-5 (PMC8907291; doi:10.1038/s41598-022-07533-5)
Supplement: Supplementary file 1 — Supplementary Information. [file 41598_2022_7533_MOESM1_ESM.pdf]

Experimental Section

All glassware was oven-dried prior to use. Under otherwise noted, solvents and reagents were obtained from commercial suppliers (Sigma Aldrich, TCI, Carlo Erba, Acros, Merck) and used without further purification. Thin layer chromatography (TLC) was performed using silica gel 60 F254 (Merck) and visualized under UV light. Column chromatography was performed on silica gel (mesh 300-400). ^{1}H NMR and ^{13}C NMR spectra were recorded on a Bruker Avance 500 MHz spectrometer at room temperature in MeOD with Me_4Si as an internal standard. Chemical shifts of ^{1}H NMR spectra were recorded and reported in ppm from the solvent resonance (MeOD at 3.34 ppm). Data were reported as follows: chemical shift in ppm (δ), multiplicity (s = singlet, d = doublet, t = triplet, q = quartet, quin = quintet, br = broad and m = multiplet), coupling constant in Herts (Hz), integration and only major peaks are reported in cm^{-1}. ^{13}C NMR spectra were also recorded in ppm from the solvent resonance (MeOD at 49.86 ppm). HRMS and mass data were recorded by ESI on a TOF mass spectrometer.

General procedure for the preparation of IR794

1. Synthetic procedures

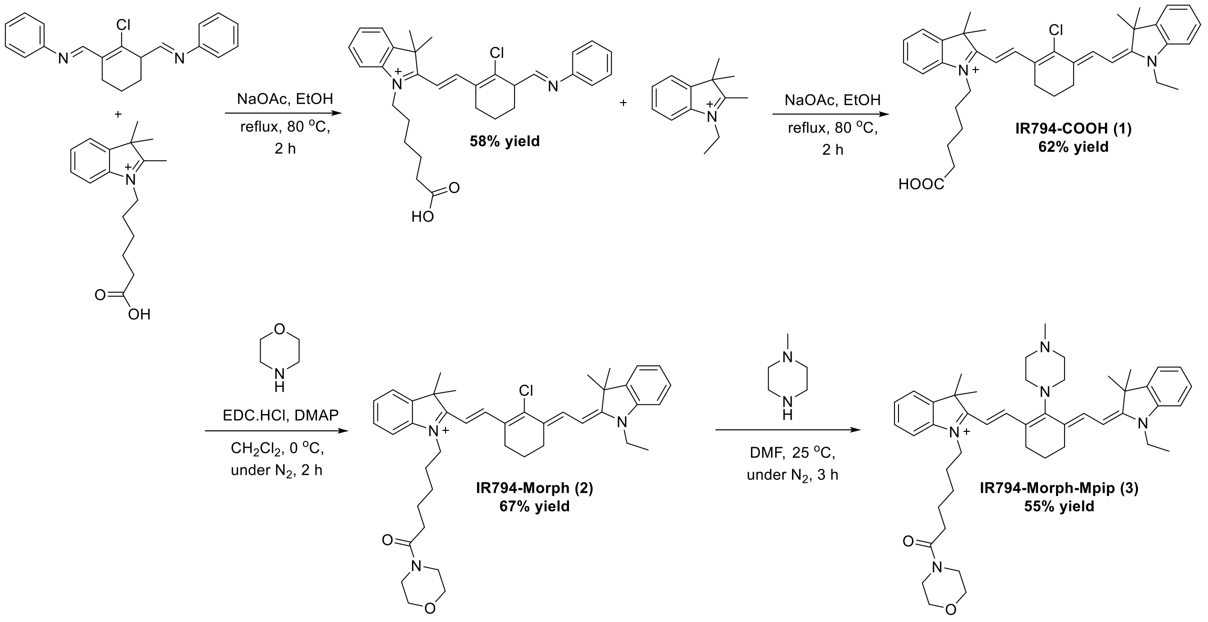

2. Syntheses of IR794 derivatives

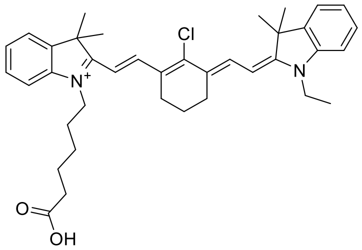

1-(5-carboxypentyl)-2-((*E*)-2-((E)-2-chloro-3-(2-((*E*)-1-ethyl-3,3-dimethylindolin-2 ylidene)ethylidene)cyclohex-1-en-1-yl)vinyl)-3,3-dimethyl-3*H*-indol-1-ium (1)

IR794-COOH, (1*E*,1'*E*)-1,1'-(2-chlorocyclohex-1-ene-1,3-diyl)bis(*N*-phenylmethanimine) (300 mg, 0.60 mmol) and 6-(2,3,3-trimethyl-3*H*-1λ^4-indol-1-yl)hexanoic acid (180 mg, 0.60 mmol) and anhydrous sodium acetate (59 mg, 0.72 mmol) were heated at 80 ºC for 2 h in EtOH (5 mL). The solvent was removed and purified by silica gel column chromatography (2% v/v MeOH/CH_2Cl_2) to give the intermediate (1-(5-carboxypentyl)-2-((*E*)-2-(2-chloro-3-((*E*)-(phenylimino)methyl)cyclohex-1-en-1-yl)vinyl)-3,3-dimethyl-3*H*-indol-1-ium, asymetry) as a blue solid (257 mg, 85% yield). And then used the intermediate (257 mg, 0.51 mmol), 1-ethyl-2,3,3-trimethyl-3*H*-1λ^4-indole (96 mg, 0.51 mmol), and anhydrous sodium acetate (50 mg, 0.61 mmol) were heated at 80 ºC for 2 h in EtOH (5 mL). The solvent was removed and the crude product was then purified by silica gel column chromatography (5% v/v MeOH/CH_2Cl_2) to yield the product as a green solid (189 mg, 62% yield). Characterization of **IR794-COOH**; HRMS: m/z $[Na]^+$calc for $C_{38}H_{45}ClN_2NaO_2^+$: 619.3077; found: 619.3062. ^{1}H NMR (500 MHz, MeOD) *δ* 8.49 (dd, *J* = 14.1, 11.8 Hz, 2H), 7.61 (t, *J* = 7.1 Hz, 2H), 7.51 (q, *J* = 7.3 Hz, 2H), 7.46 – 7.40 (m, 2H), 7.36 (q, *J* = 7.9 Hz, 2H), 6.38 (dd, *J* = 16.2, 14.1 Hz, 2H), 4.33 (q, *J* = 7.2 Hz, 2H), 4.27 (t, *J* = 7.5 Hz, 2H), 2.82 (q, *J* = 5.2 Hz, 4H), 2.40 (t, *J* = 7.3 Hz, 2H), 2.03 (quin, *J* = 6.3 Hz, 2H), 1.94 (quin, *J* = 7.4 Hz, 2H), 1.80 (s, 12H), 1.60 (q, *J* = 8.3 Hz, 2H), 1.50 (t, *J* = 7.2 Hz, 3H), 1.43 (d, *J* = 7.4 Hz, 2H), ^{13}C NMR (125 MHz, MeOD) *δ* 176.0, 172.6, 172.5, 149.6, 144.3, 143.8, 142.2, 141.7, 141.4, 141.2, 128.6, 128.6, 126.7, 126.6, 125.3, 125.1, 122.3, 122.2, 110.9, 110.8, 100.9, 53.6, 49.3, 49.2, 43.9, 39.2, 33.5, 27.1, 27.0, 26.8, 26.2, 26.1, 26.0, 24.4, 20.7, 11.4.

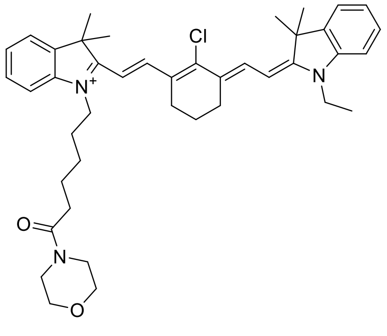

2-((*E*)-2-((*E*)-2-chloro-3-(2-((*E*)-1-ethyl-3,3-dimethylindolin-2-ylidene)ethylidene)cyclohex-1-en-1-yl)vinyl)-3,3-dimethyl-1-(6-morpholino-6-oxohexyl)-3*H*-indol-1-ium (2)

IR794-Morph, to a stirred and cooled (0 °C) solution of the **IR794-COOH** (70 mg, 0.117 mmol) in CH_2Cl_2 (3 mL), EDC.HCl (45 mg, 0.234 mmol) and DMAP (7 mg, 0.058 mmol) were added and continued stirring for 30 min. Morpholine (15 mg, 0.14 mmol) in CH_2Cl_2 (2 mL) was then added to the

solution and continued stirring for 3 h at 25 °C, under nitrogen atmosphere. The crude product was then purified by silica gel column chromatography (5% v/v MeOH/CH_2Cl_2) to yield a green solid as a product (52 mg, 67% yield). Characterization of **IR794-Morph**; HRMS: m/z $[M]^+$calc for $C_{42}H_{53}ClN_3O_2^+$: 666.3830; found: 666.3821. ^{1}H NMR (500 MHz, MeOD) *δ* 8.53 (dd, *J* = 14.5, 12.0 Hz, 2H), 7.62 (t, *J* = 6.0 Hz, 2H), 7.56 – 7.50 (m, 2H), 7.44 (d, *J* = 8.0 Hz, 2H), 7.38 (q, *J* = 7.5 Hz, 2H), 6.39 (dd, *J* = 14.0, 9.5 Hz, 2H), 4.32 (dt, *J* = 14.5, 7.5 Hz, 4H), 3.70 (dt, *J* = 10.0, 5.0 Hz, 4H), 3.61 (dt, *J* = 10.0, 4.5 Hz, 4H), 2.84 (t, *J* = 6.0 Hz, 4H), 2.51 (t, *J* = 7.5 Hz, 2H), 2.05 (quin, *J* = 5.5 Hz, 2H), 1.97 (quin, *J* = 7.5 Hz, 2H), 1.82 (s, 12H), 1.77 (quin, *J* = 7.5 Hz, 2H), 1.63 – 1.55 (m, 2H), 1.51 (t, *J* = 7.0 Hz, 3H), ^{13}C NMR (125 MHz, MeOD) *δ* 172.7, 172.6, 172.5, 149.7, 144.4, 143.9, 142.3, 141.7, 141.4, 141.2, 128.6, 128.5, 126.6, 126.5, 125.3, 125.1, 122.2, 122.2, 111.0, 110.8, 100.9, 100.8, 66.5, 66.4, 53.5, 49.3, 49.2, 46.0, 43.8, 41.9, 39.1, 32.0, 27.0, 26.9, 26.1, 26.1, 26.0, 24.7, 20.8, 11.2.

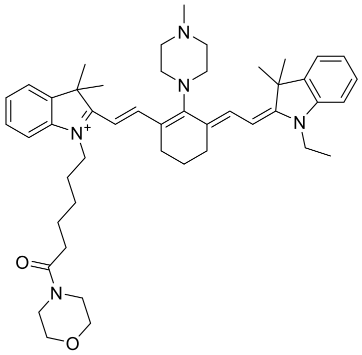

2-((*E*)-2-((*E*)-3-(2-((*E*)-1-ethyl-3,3-dimethylindolin-2-ylidene)ethylidene)-2-(4-methylpiperazin-1-yl)cyclohex-1-en-1-yl)vinyl)-3,3-dimethyl-1-(6-morpholino-6-oxohexyl)-3*H*-indol-1-ium

IR794-Morph-Mpip used **IR794-Morph** (60 mg, 0.09 mmol) was dissolved in anhydrous DMF (3.0 mL), then *N*-methylpiperazine (8.92 mg, 0.09 mmol) was added to the solution. Thereafter, the mixture was stirred at 25 °C for 3 h under nitrogen atmosphere. After that, the solvent was removed under reduced pressure and the crude product was purified by column chromatography using CH_2Cl_2:MeOH (gradient from ratio 9:1 to 7:3) as the eluent to afford a blue solid (35 mg, 55% yield). Characterization of **IR794-Morph-Mpip**: HRMS: m/z $[M]^+$calc for $C_{47}H_{64}N_5O_3^+$: 730.5161; found: 730.5055. ^{1}H NMR (500 MHz, MeOD) *δ* 7.90 (dd, *J* = 13.5, 7.0 Hz, 2H), 7.54 (dd, *J* = 7.0, 2.5 Hz, 2H), 7.49 – 7.42 (m, 2H), 7.31 – 7.24 (m, 4H), 6.10 (dd, *J* = 13.5, 4.0 Hz, 2H), 4.16 (dt, *J* = 22.0, 7.0 Hz, 4H), 3.85 (t, *J* = 4.5 Hz, 4H), 3.75 – 3.67 (m, 4H), 3.63 (t, *J* = 5.0 Hz, 2H), 3.59 (t, *J* = 5.0 Hz, 2H), 2.87 (t, *J* = 4.5 Hz, 4H), 2.64 (td, *J* = 6.5, 3.5 Hz, 4H), 2.59 (s, 3H), 2.50 (t, *J* = 7.0 Hz, 2H), 1.93 (td, *J* = 12.5, 6.0 Hz, 4H), 1.79 (d, *J* = 2.0 Hz, 12H), 1.75 (t, *J* = 8.0 Hz, 2H), 1.57 (q, *J* = 8.5 Hz, 2H), 1.45 (t, *J* = 7.0 Hz, 3H), ^{13}C NMR (125 MHz, MeOD) *δ* 172.7, 172.5, 169.5, 169.3, 142.7, 142.2, 142.0, 141.6, 140.6, 140.4, 128.3, 128.2, 124.6, 124.5, 123.6, 123.5, 121.9, 121.8, 109.6, 109.3, 96.4, 96.0, 66.5, 66.4, 56.3, 54.1, 46.0, 45.0, 42.9, 41.9, 38.0, 32.0, 27.9, 27.7, 26.5, 26.2, 24.7, 24.6, 24.5, 21.7, 10.6.

3. NMR Spectra of IR794 derivatives

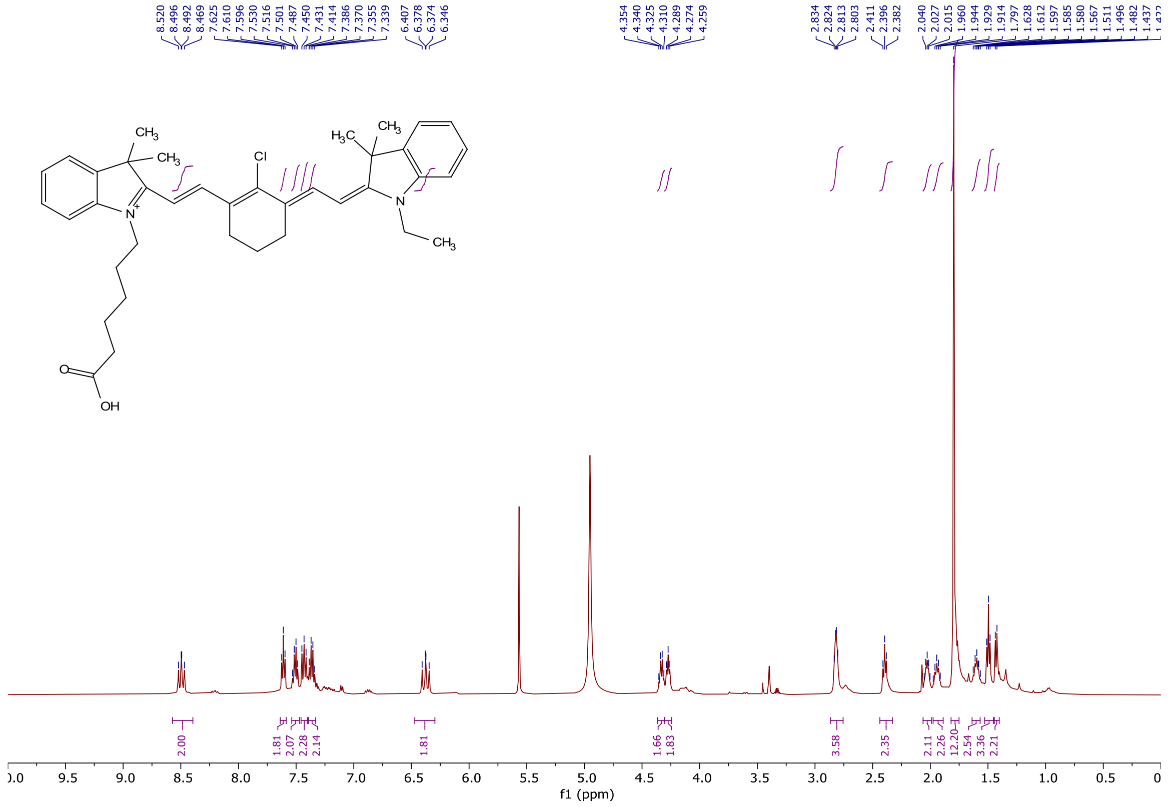

^{1}H NMR of **IR794-COOH**

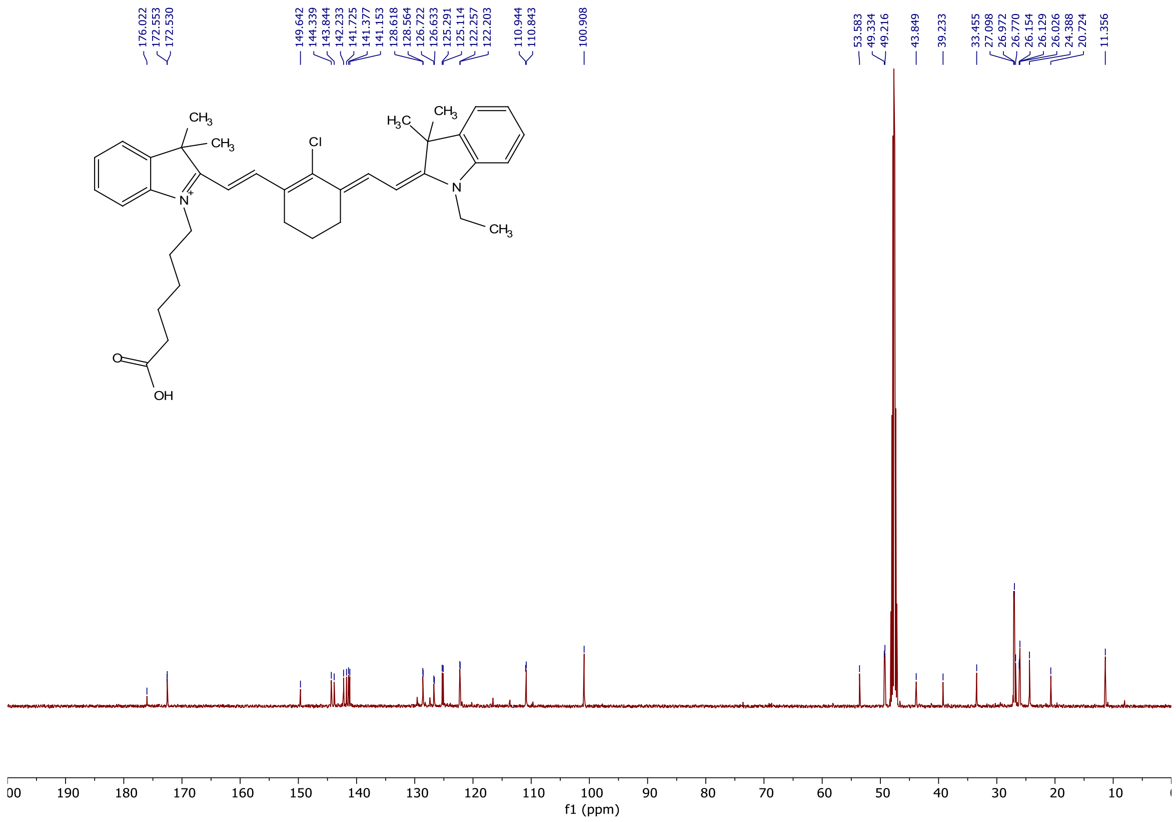

^{13}C NMR of **IR794-COOH**

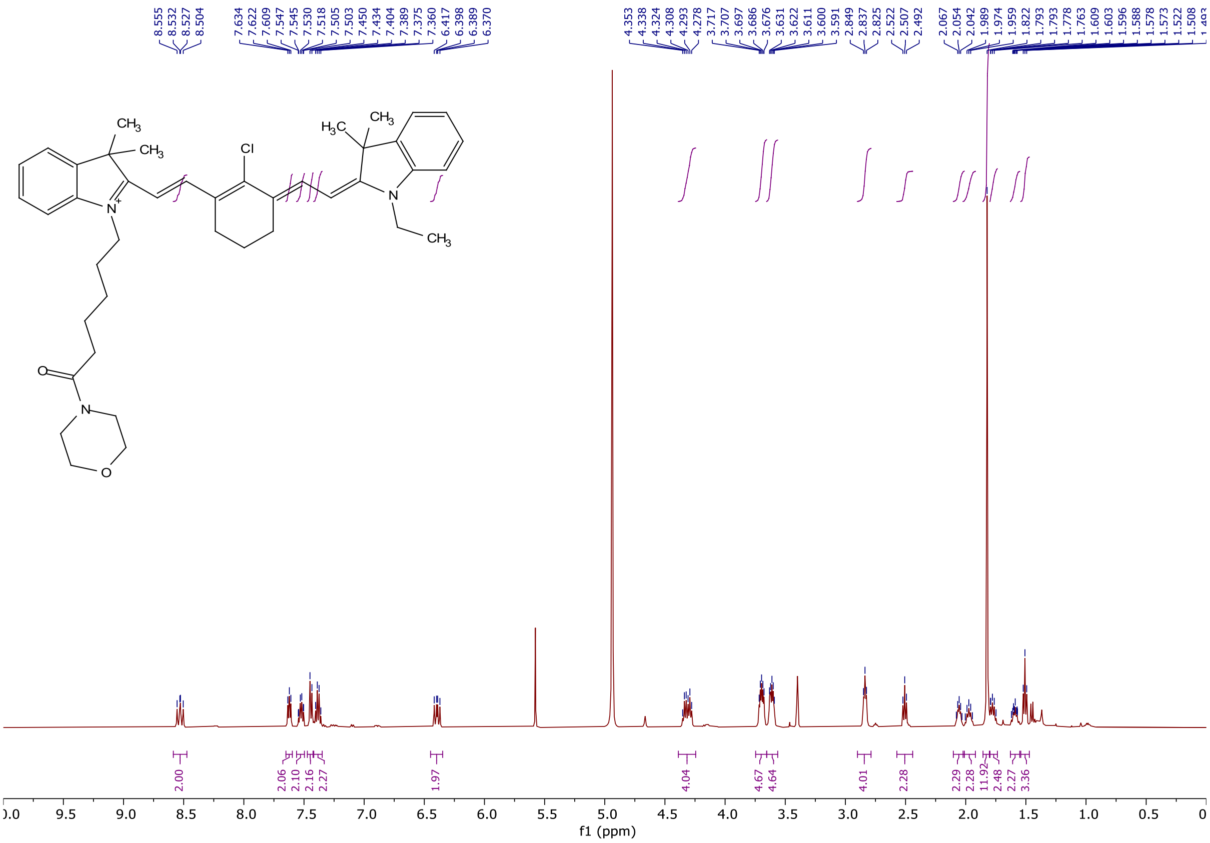

^{1}H NMR of **IR794-Morph**

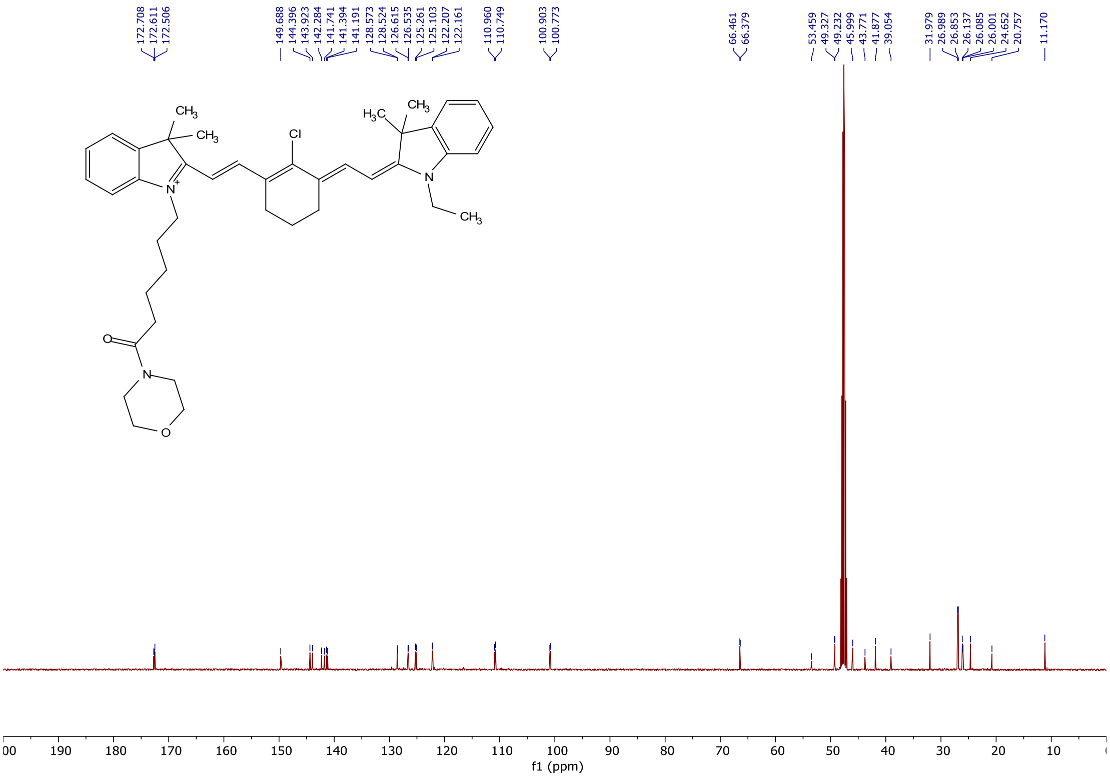

^{13}C NMR of **IR794-Morph**

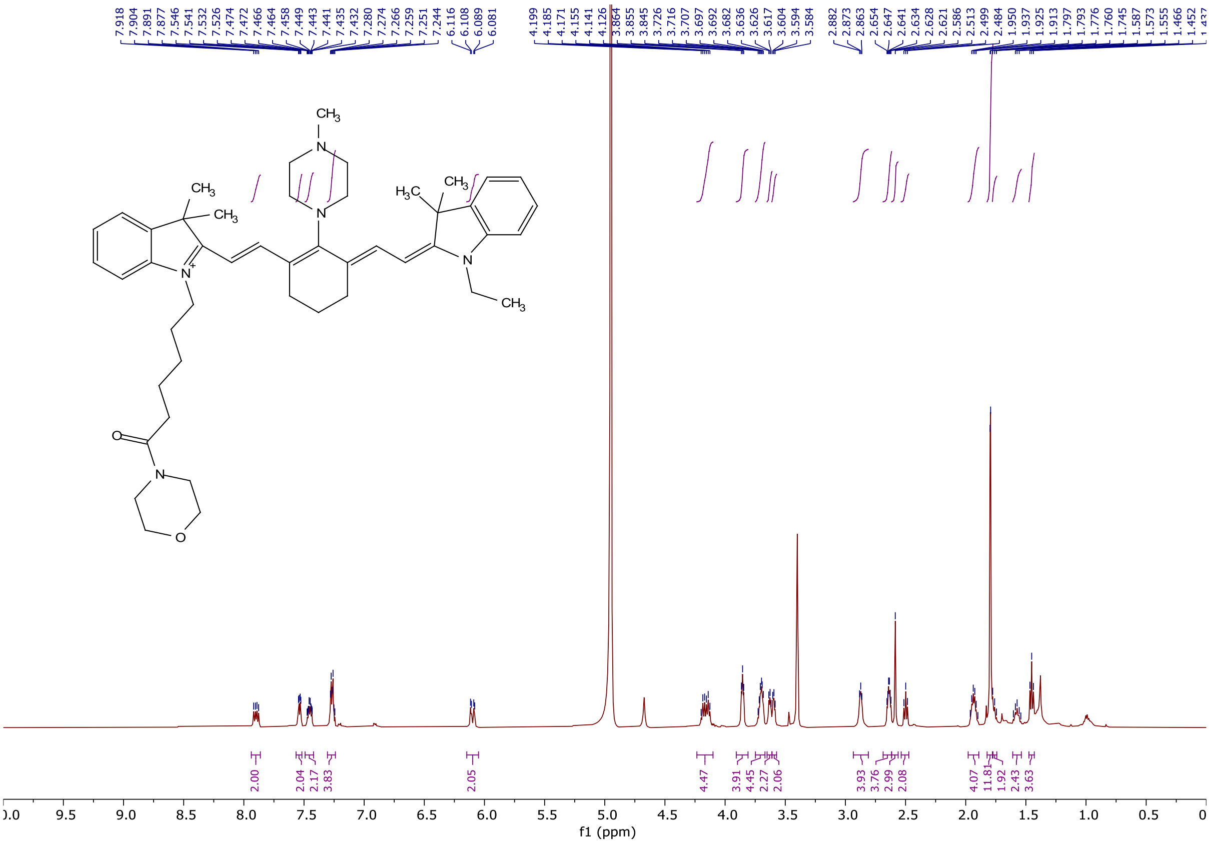

^{1}H NMR of **IR794-Morph-Mpip**

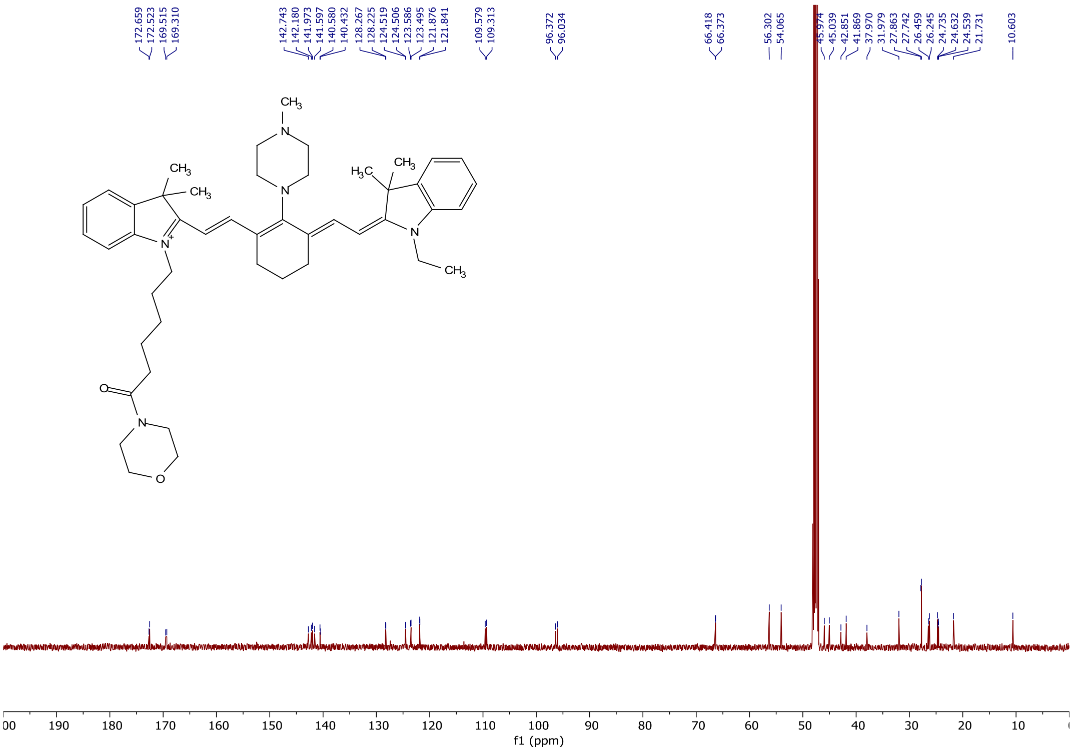

^{13}C NMR of **IR794-Morph-Mpip**

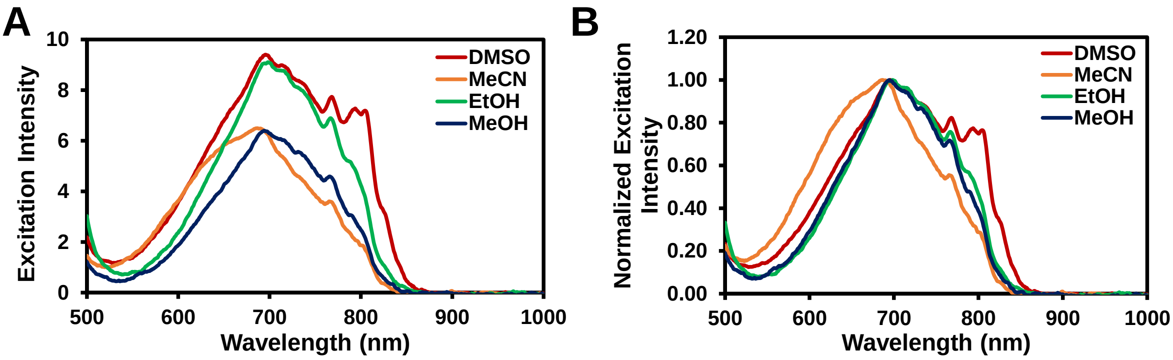

Figure S1. Excitation spectra (**A**) and normalized intensity (**B**) of **IR794-Morph-Mpip** in various solvents.

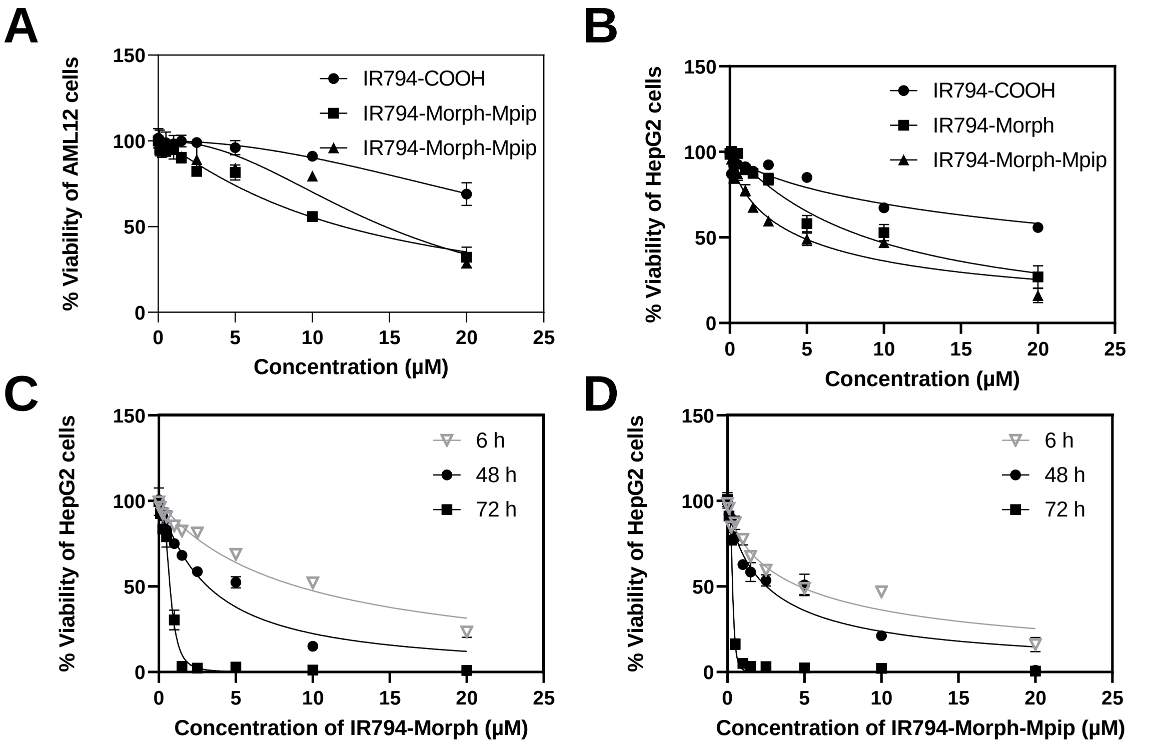

Figure S2. IC_{50} of **IR794** derivatives incubated with (**A**) AML12 and (**B**) HepG2 cells for 6 h. IC_{50} of **IR794-Morph** (**C**) and **IR794-Morph-Mpip** (**D**) incubated with HepG2 cells for 6, 48 and 72 h.

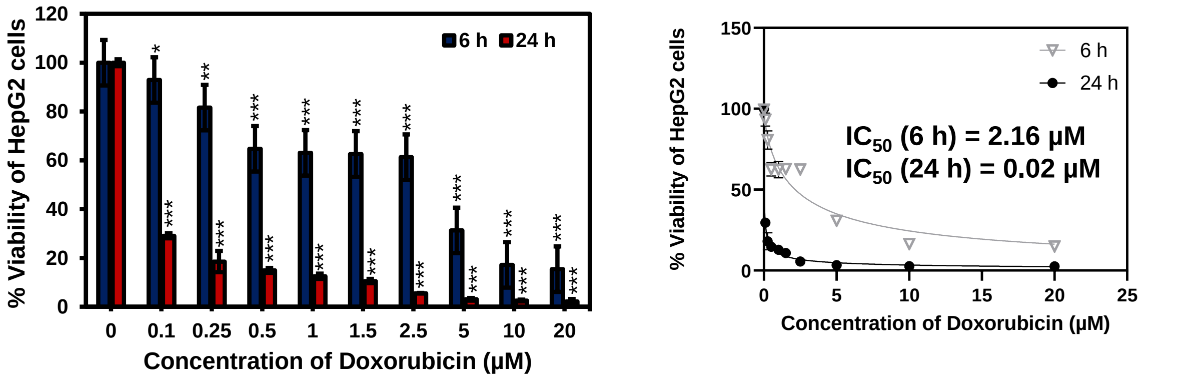

Figure S3. Relative cell viability of HepG2 treated with doxorubicin for 6 and 24 h.

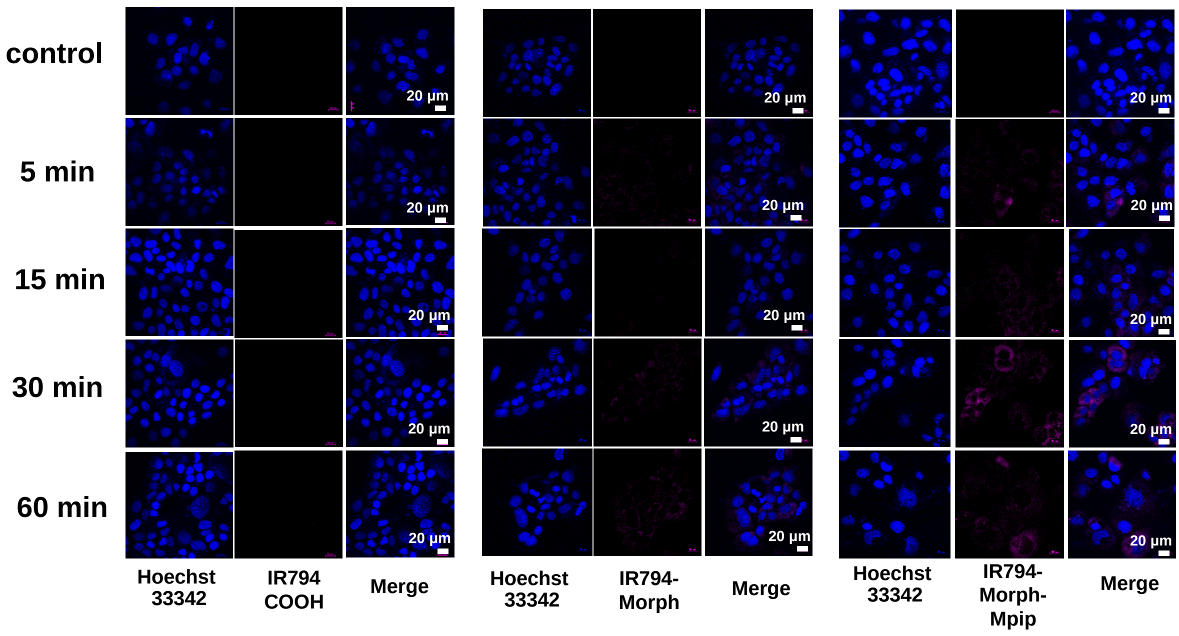

Figure S4. Time dependent internalization of AML12 cells treated with **IR794** probes (1 µM).

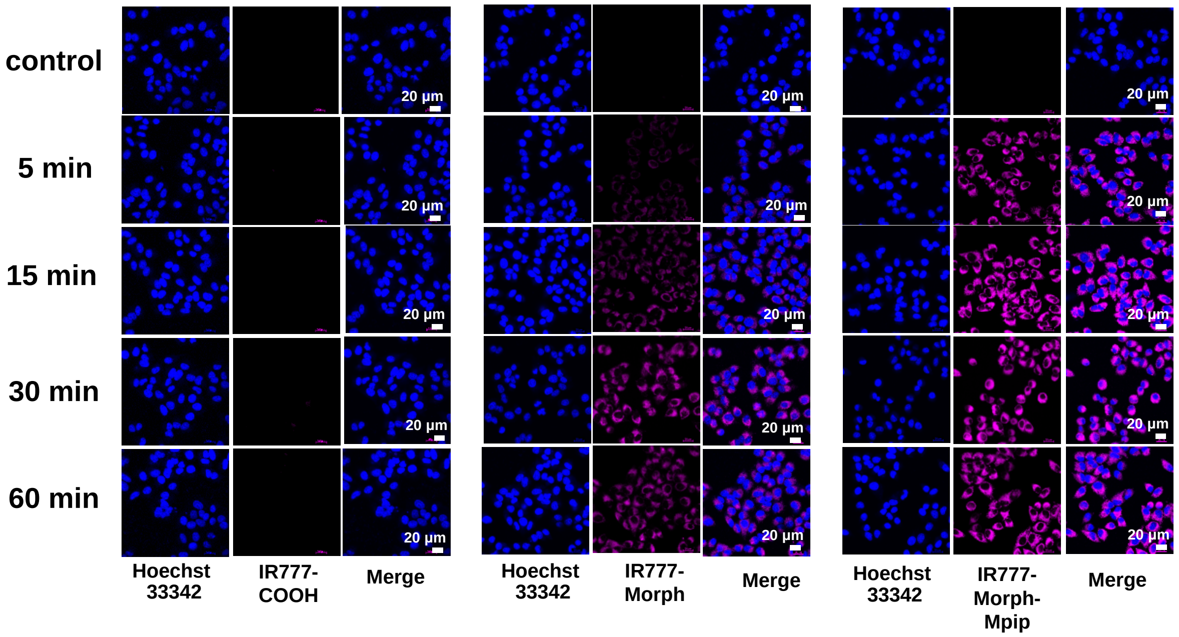

Figure S5. Time dependent internalization of HepG2 cells treated with **IR794** probes (1 µM).

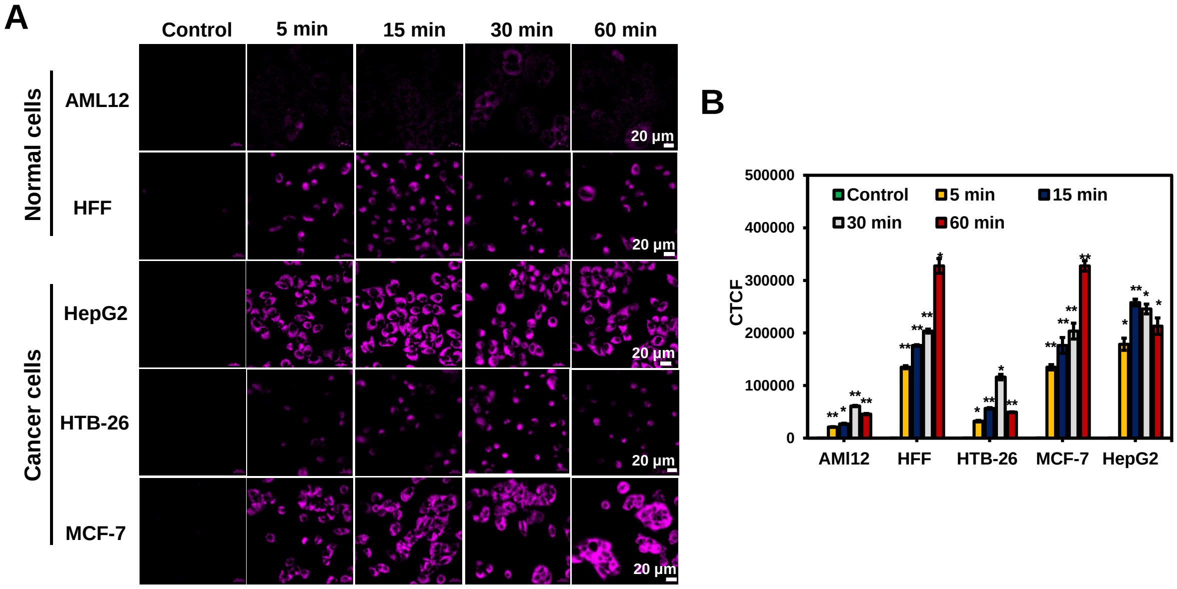

Figure S6. (**A**) Confocal images of various cell lines incubated with **IR794-Morph-Mpip** (1 µM) for different duration (5, 15, 30, and 60 min). (**B**) Quantitative corrected total cell fluorescence data, which were quantified using ImageJ and represent the mean ± SD (n = 30). Statistical analysis is based on Paired Student's T-test (*P < 0.05, **P < 0.01, ***P < 0.001).

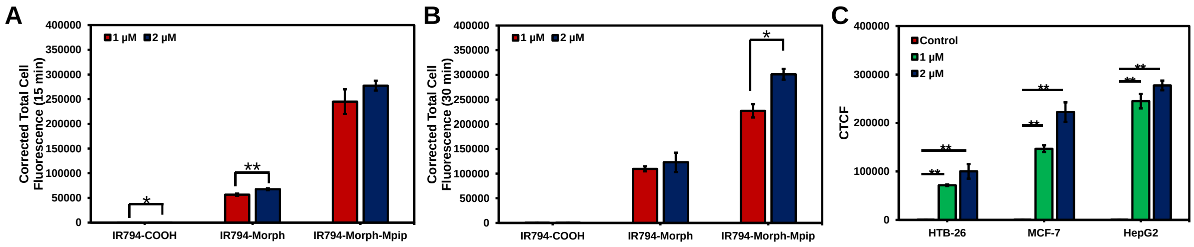

Figure S7. (**A**) Quantitative corrected total cell fluorescence (CTCF), quantified using ImageJ and represent the mean ± SD (n = 30), of HepG2 incubated with **IR794** probes at 1 and 2 µM. (B) CTCF of cancer cells incubated with **IR794-Morph-Mpip** at 1 and 2 µM.
